# Cofilactin rod formation mediates inflammation-induced neurite degeneration

**DOI:** 10.1016/j.celrep.2024.113914

**Published:** 2024-03-06

**Authors:** Gökhan Uruk, Ebony Mocanu, Alisa E. Shaw, James R. Bamburg, Raymond A. Swanson

**Affiliations:** 1 Department of Neurology, University of California, San Francisco, San Francisco, CA, USA; 2 Neurology Service, San Francisco Veterans Affairs Health Care System, San Francisco, CA, USA; 3 Department of Biochemistry and Molecular Biology, Colorado State University, Fort Collins, CO, USA; 4 Lead contact

## Abstract

Stroke, trauma, and neurodegenerative disorders cause loss of neurites (axons and dendrites) in addition to neuronal death. Neurite loss may result directly from a primary insult, secondary to parental neuron death, or secondary to a post-injury inflammatory response. Here, we use lipopolysaccharide and the alarmin S100β to selectively evaluate neurite loss caused by the inflammatory response. Activation of microglia and infiltrating macrophages by these stimuli causes neurite loss that far exceeds neuronal death, both *in vitro* and *in vivo*. Neurite loss is accompanied by the formation of cofilactin rods and aggregates (CARs), which are polymers of cofilin-1 and actin induced by oxidative stress and other factors. Mice deficient in either cofilin-1 or the superoxide-generating enzyme NADPH oxidase-2 show reduced CAR formation, neurite loss, and motor impairment. The findings identify a mechanism by which inflammation leads to neurite loss via CAR formation and highlight the relevance of neurite loss to functional impairment.

## In brief

Uruk et al. demonstrate that the brain inflammatory response causes neurite (axon and dendrite) degeneration by a mechanism involving NOX2-induced cofilactin rod formation. Neurites are more vulnerable to inflammation-induced injury than neuronal cell bodies, and neurite loss in the absence of neuronal cell body loss is associated with functional impairment.

## Graphical Abstract

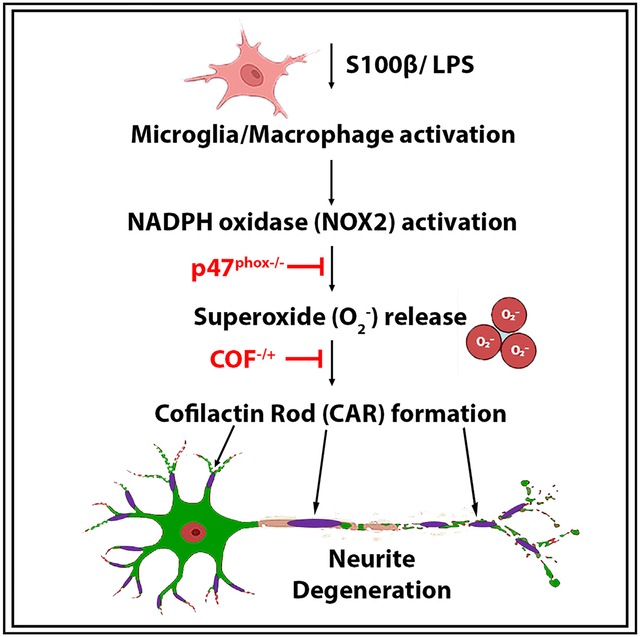

## INTRODUCTION

Brain microglia and macrophages (M/M) respond to pro-inflammatory stimuli with an innate immune response that includes the release of cytokines, chemokines, proteases, and reactive oxygen species.^1–3^ This response provides a rapid first-line reaction to infection and trauma but can itself injure neighboring neurons and other cell types. The capacity for this response to cause neuronal death is well established and has been shown to contribute to a variety of neurological disorders, including Alzheimer’s disease, Parkinson’s disease, and secondary injury following stroke and brain trauma.^1,4–6^ By contrast, far less is known about inflammation-induced injury to neurites, i.e., neuronal axons and dendrites.^3^

Neurite degeneration can result from the formation of rod-shaped bundles of cofilin-saturated F-actin, termed cofilactin rods (CARs), as well as other cofilin-containing aggregates.^7–10^ Cofilin-1, the ubiquitous mammalian member of the actin-depolymerizing factor-cofilin family, is best known for regulating the dynamic turnover of actin filaments by severing and depolymerizing F-actin. However, cofilin-1 also binds cooperatively along F-actin and can lead to stable cofilactin filaments, which subserve cytoskeletal functions such as the formation of growth cone filopodia.^11,12^ The events leading to pathological CAR formation involve the activation of cofilin-1 by dephosphorylation of its serine-3 residue and the formation of cofilin dimers through intermolecular disulfide bonds.^13–15^ CAR formation causes local disruption of cytoskeletal dynamics in neurites, such as anterograde and retrograde trafficking.^16–18^ Importantly, this process can occur in the absence of parent neuron demise.

CARs can be identified by immunostaining as focal, rod-shaped aggregates of cofilin-1 and have been observed in animal models of Alzheimer’s disease, stroke, and other conditions.^10,18,19^ More amorphous coflin-1-containing aggregates are also observed under conditions that form CARs and may represent higher-order polymers, CARs rendered extracellular after neurite degeneration, or other types of cofilin-1 aggregates.^10,20^ In fixed brain tissue, it is often difficult to colocalize CARs and CAR-related aggregates exclusively to neurites because the cytoskeleton markers for neurites are lost at sites of CAR formation, but in cell culture studies, it is clear that these form in neuronal processes and, to a lesser extent, in neuronal cell bodies.^20,21^ CARs and related cofilin-1 aggregates induced by brain ischemia do not colocalize with CNPase, GFAP, CD11b, or CD31, suggesting that they do not form in brain oligodendrocytes, astrocytes, M/M, or endothelium.^21^

A potential link between the innate immune response and CAR formation is the oxidative stress produced by activated microglia and infiltrating macrophages (M/M).^22^ M/M and other immune cells markedly increase superoxide and nitric oxide production in response to pro-inflammatory stimuli.^23,24^ These stimuli include both exogenous factors such as bacterial lipopolysaccharide (LPS) and the release of endogenous compounds termed “alarmins,” such as the normally intracellular proteins S100β and HMGB1.^25–29^ The production of superoxide and nitric oxide leads in turn to oxidative stress through the formation of highly reactive oxygen species such as hydrogen peroxide, peroxynitrite, and hydroxyl radical. Oxidative stress drives CAR formation in two ways: by activating phosphatases that dephosphorylate cofilin-1 at its serine-3 residue and by forming disulfide linkages between cofilin-1 molecules.^21,22,30^

These factors suggest that the innate immune response might be a factor driving CAR formation and neurite degeneration. Here, we evaluated this possibility in mouse cortex and primary cultures. Our findings show that inflammation causes neurite degeneration far out of proportion to neuronal death and that the neurite degeneration results from CAR formation in response to M/M superoxide production.

## RESULTS

### S100β induces CAR formation and subsequent neurite loss

**Figure 1.**
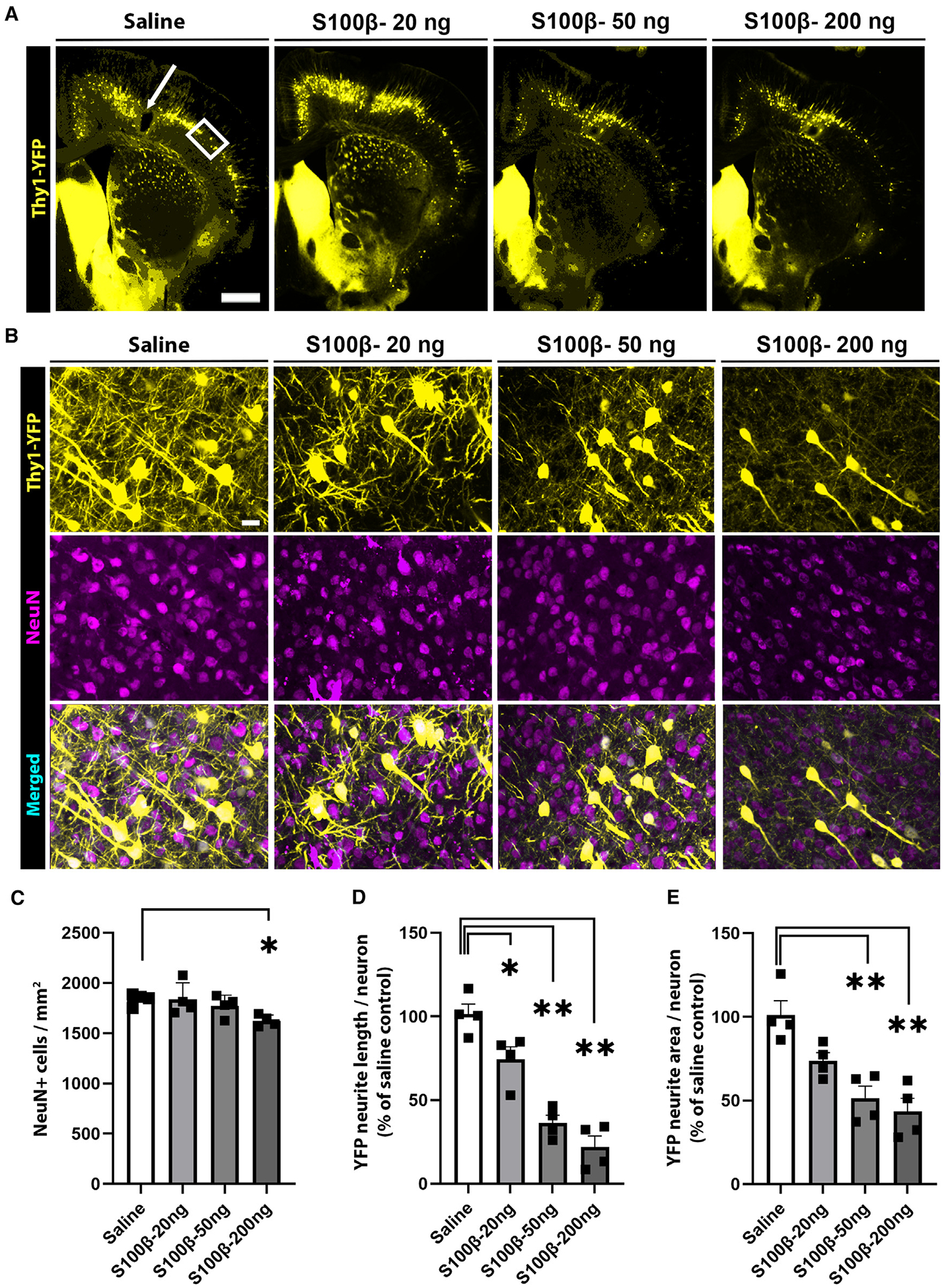
S100β induces neurite loss (A and B) Low- and high-magnification views of brain sections from Thy1-YFP mice harvested 3 days after S100β injection. Scale bar: 500 μm. In (A), the arrow shows the injection site, and the rectangle shows the area of higher-magnification views shown in (B). In (B), neuronal cell nuclei are identified by NeuN immunostaining (magenta). Scale bar: 10 μm. (C) Quantification of neuronal cell body density. (D and E) YFP neurite length and area, expressed relative to saline-injected controls. n = 4; *p < 0.05 and **p < 0.01 vs. saline controls by one-way ANOVA with Dunnett’s test. All data are shown as mean ± SEM.

We used Thy1-YFP mice to visualize neuronal cell bodies, axons, and dendrites of mice in which the alarmin S100β was stereotaxically injected into the primary motor cortex. These mice developed a dose- and time-dependent loss of neurites, as assessed by measures of both neurite length and neurite area (Figure 1). By contrast, there was near-complete sparing of neuronal cell bodies over the range of S100β doses used (Figure 1C). Neuronal cell body survival was also confirmed in a separate set of experiments with wild-type (WT) mice, using NeuN to identify neuronal cell nuclei. In these studies, there was no discernible loss of neuronal nuclei at time points up to 2 weeks after S100β injections (Figure S1A). There was also no detectable reduction in neuronal nuclear size, as would be expected with incipient cell death^31,32^ (Figures S1B–S1F).

**Figure 2.**
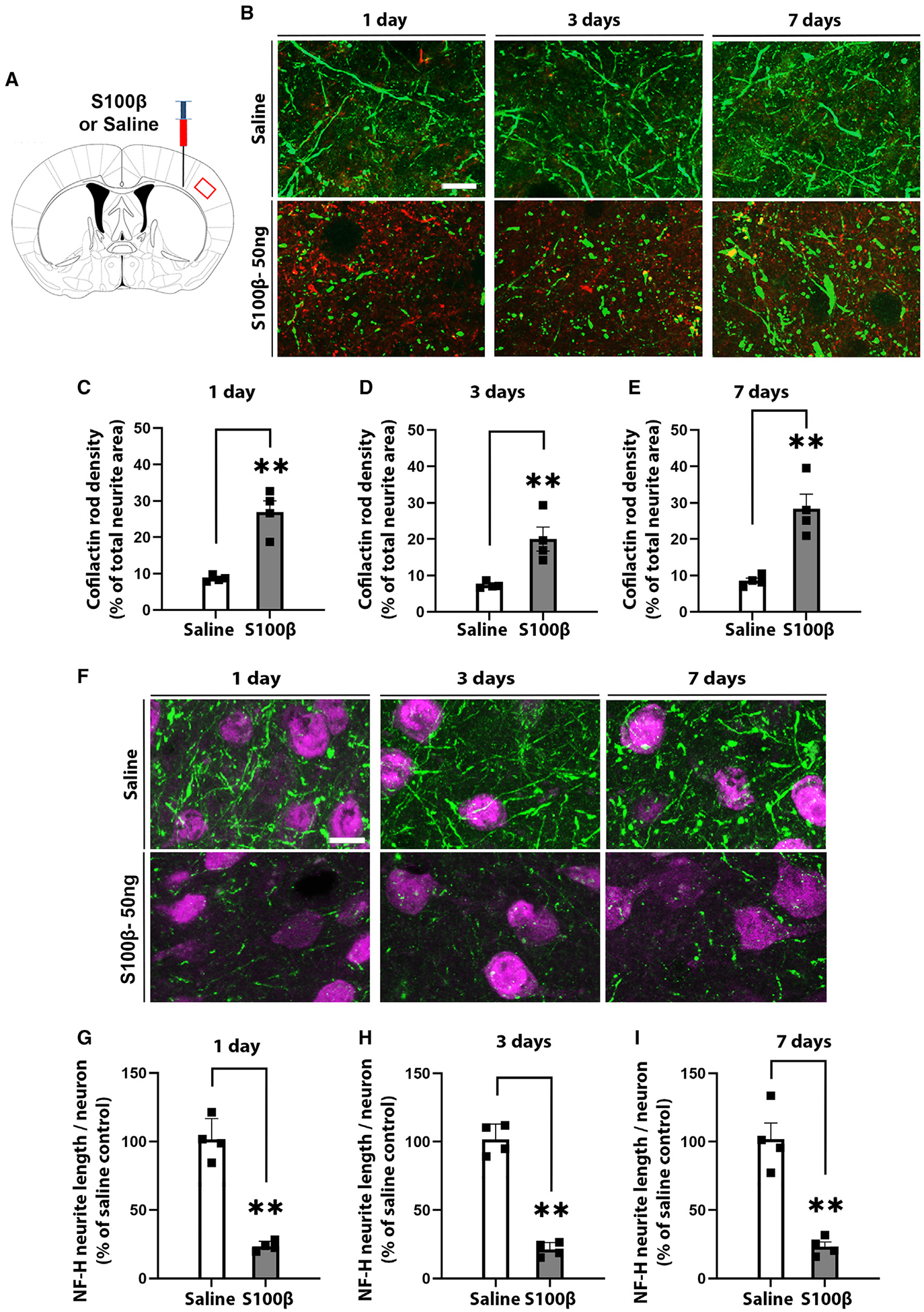
S100β induces cofilactin rod (CAR) formation and neurite loss (A) Diagram shows the locations of intracortical injections and peri-injection region imaged. (B) CAR formation identified by immunostaining for cofilin-1 aggregates (red) in WT mice after saline or 50 ng S100β injections. Neurites are identified by neurofilament-H (NF-H; green). NF-H integrity is lost at sites of CAR formation. Scale bar: 10 μm. (C–E) CAR density expressed as percentage of total neurite area. (F) Neurite loss 1, 3, and 7 days after saline or 50 ng S100β injections as assessed by immunostaining for NF-H (green), with neuronal cell nuclei identified by NeuN (magenta). Scale bar: 10 μm. (G–I) NF-H+ neurite length per neuronal nucleus in the S100β-injected mice. n = 4; **p < 0.01 by Student’s t test. All data are shown as mean ± SEM.

To determine if the neurite loss was associated with the formation of CARs, we used immunostaining for aggregated cofilin-1.^16,21^ In these and subsequent studies, we used WT rather than YFP-expressing mice because the broad fluorescence emission of YFP limits the use of other fluorescent markers. Focal, rod-like aggregates of cofilin-1 indicative of CAR formation were observed in the brains injected with S100β, along with other, more amorphous cofilin-1 aggregates (Figures 2A and 2B). As previously described, neurofilament integrity was lost at sites of CAR formation, and the CARs often formed linear patterns corresponding to the lost or degenerating neurites.^20,21^ CAR formation was maximal by 1 day after the S100β injections (Figures 2B–2E) and was followed by subsequent neurite loss as assessed by both total neurite length (Figures 2F–2I) and neurite area (Figure S2). Mice injected with 50 ng denatured (boiled) S100β did not exhibit any increased CAR formation, neurite loss, or M/M activation relative to saline-injected controls (Figure S3).

### CAR formation and neurite loss is attenuated in cofilin-1 hemizygous and p47^phox^-deficient mice

**Figure 3.**
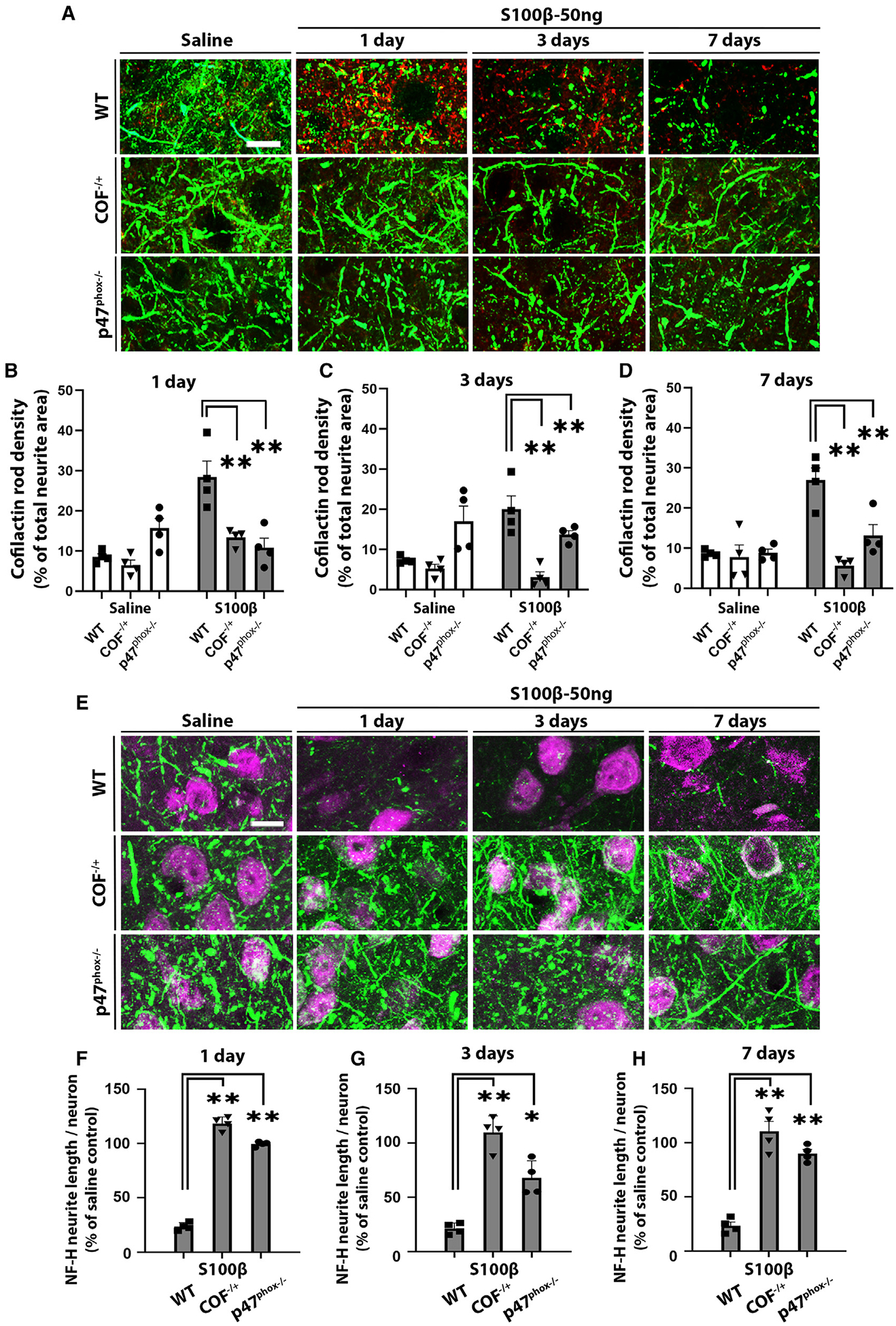
CAR formation is attenuated in both cofilin-1 hemizygous and p47^phox^-deficient mice (A) CAR formation after saline or 50 ng S100β injections in WT, *COF*^−/+^, and *p47*^phox−/−^ mice identified by immunostaining for cofilin-1 aggregates (red). Neurites are identified by NF-H (green). Scale bar: 10 μm. (B–D) CAR density expressed as percentage of total neurite area. (E) Neurite loss after saline or 50 ng S100β injections as assessed by immunostaining for NF-H (green), with neuronal cell nuclei identified by NeuN (magenta). Scale bar: 20 μm. (F–H) Neurite length per neuronal nucleus in the S100β-injected mice relative to saline-injected mice of each genotype. n = 4; *p < 0.05 and **p < 0.01 vs. WT mice by one-way ANOVA with Dunnett’s test. All data are shown as mean ± SEM.

To confirm a causal relationship between CAR formation and neurite loss, we employed cofilin-1 hemizygous (*COF*^−/+^) mice, which express reduced levels of cofilin-1 (Figure S4) and consequently have reduced propensity for CAR formation.^33^ *COF*^−/+^ mice injected with S100β showed strikingly attenuated formation of CARs (Figures 3A–3D). Importantly, these mice also showed no detectable loss of neurites (Figures 3E–3H), supporting a requisite role for CARs in the inflammation-induced neurite degeneration.

NADPH oxidase-2 (NOX2) is the major source of superoxide released by M/M and thus is a likely source of the oxidative stress driving CAR formation. We therefore compared the effects of S100β injection in WT and *p47*^phox−/−^ mice, which cannot form an active NOX2 complex. Neuronal oxidative stress was confirmed by immunostaining for γH2AX, which accumulates at foci of DNA strand breaks, in neurons near activated M/M in the WT but not *p47*^phox−/−^ mice (Figure S5). As expected, the *p47*^phox−/−^ mice also showed both reduced CAR formation and neurite loss relative to WT mice (Figure 3).

### Functional impairment caused by S100β injection is attenuated in both *COF*^−/+^ and *p47*^phox−/−^ mice

**Figure 4.**
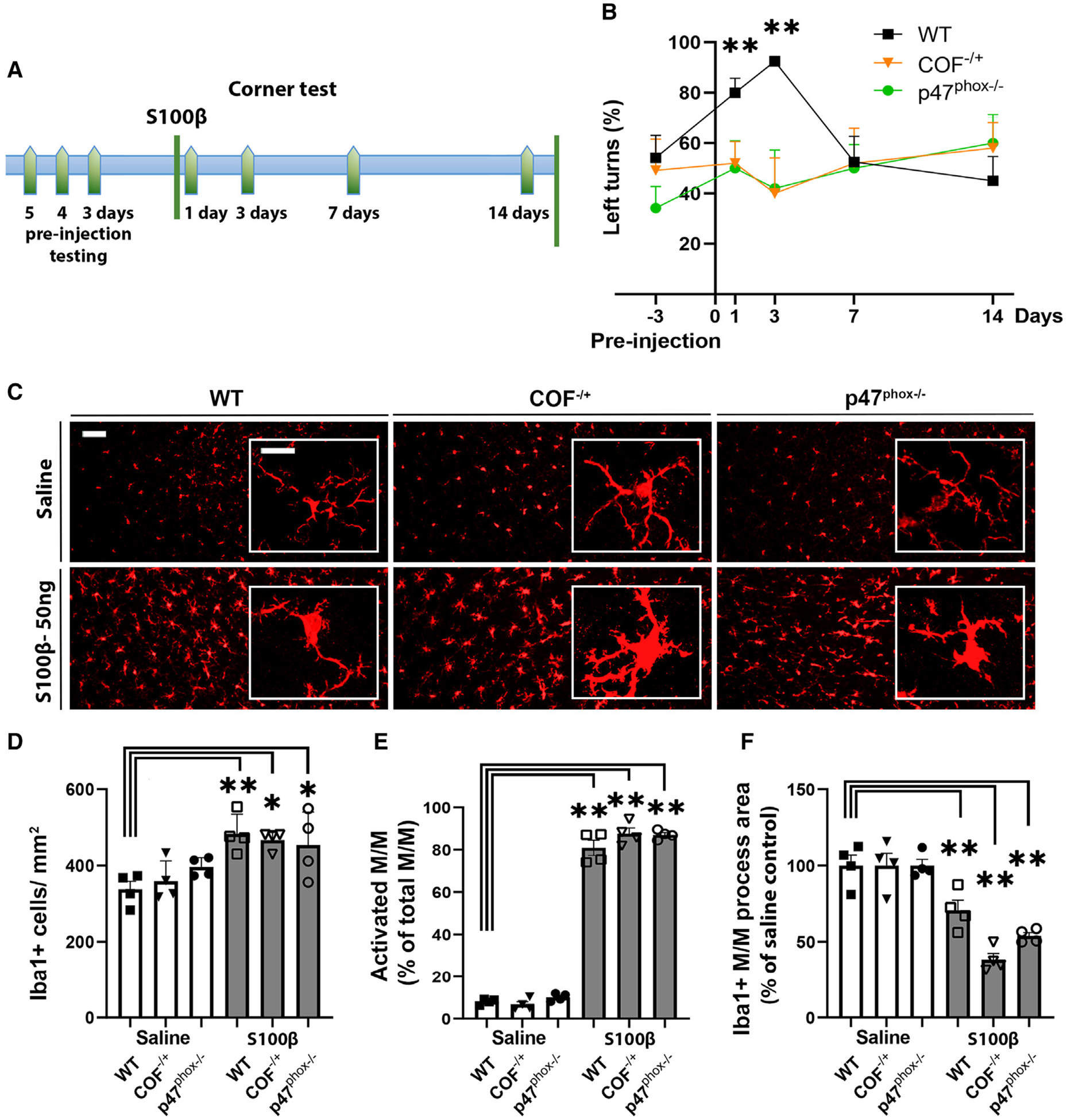
S100β-induced neurite loss and motor impairment are attenuated in cofilin-1 hemizygous and p47^phox^-deficient mice (A) Schematic timeline of behavioral assessments. (B) Performance on the corner test assessed by quantifying the percentage of left turns. n = 6 for each genotype. *p < 0.05 and **p < 0.01 vs. WT by one-way ANOVA with Dunnett’s test. (C) Iba-1 immunostaining shows microglial/macrophage (M/M) activation 1 day after injection with 50 ng S100β. Insets show magnified views. Responses to S100β injections were similar in the three mouse genotypes. Scale bars: 10 μm. (D–F) Quantification of M/M responses to S100β. n = 4; *p < 0.05 and **p < 0.01 vs. WT mice by one-way ANOVA with Dunnett’s test. All data are shown as mean ± SEM.

Motor dysfunction in mice following S100β injections into cortex was evaluated using the corner test, which has been widely adopted as a sensitive measure of mouse forelimb impairment.^34^ These studies used the same S100β injection dose (50 ng) and location (right primary motor cortex) as used in the histology studies. WT mice showed an increased propensity to turn to the left after S100β, but not vehicle (saline), injections, with normalization by day 7 after the injection. By contrast, neither the *COF*^−/+^ or *p47*^phox−/−^ mice exhibited motor impairment at any time point evaluated (Figures 4A and 4B). Importantly, both the *COF*^−/+^ mice and *p47*^phox−/−^ mice showed the same robust M/M response to S100β injections as observed in WT mice (Figures 4C–4F), and in all three mouse genotypes, there was no negligible neuronal loss after the 50 ng S100β injections (Figure S1).

**Figure 5.**
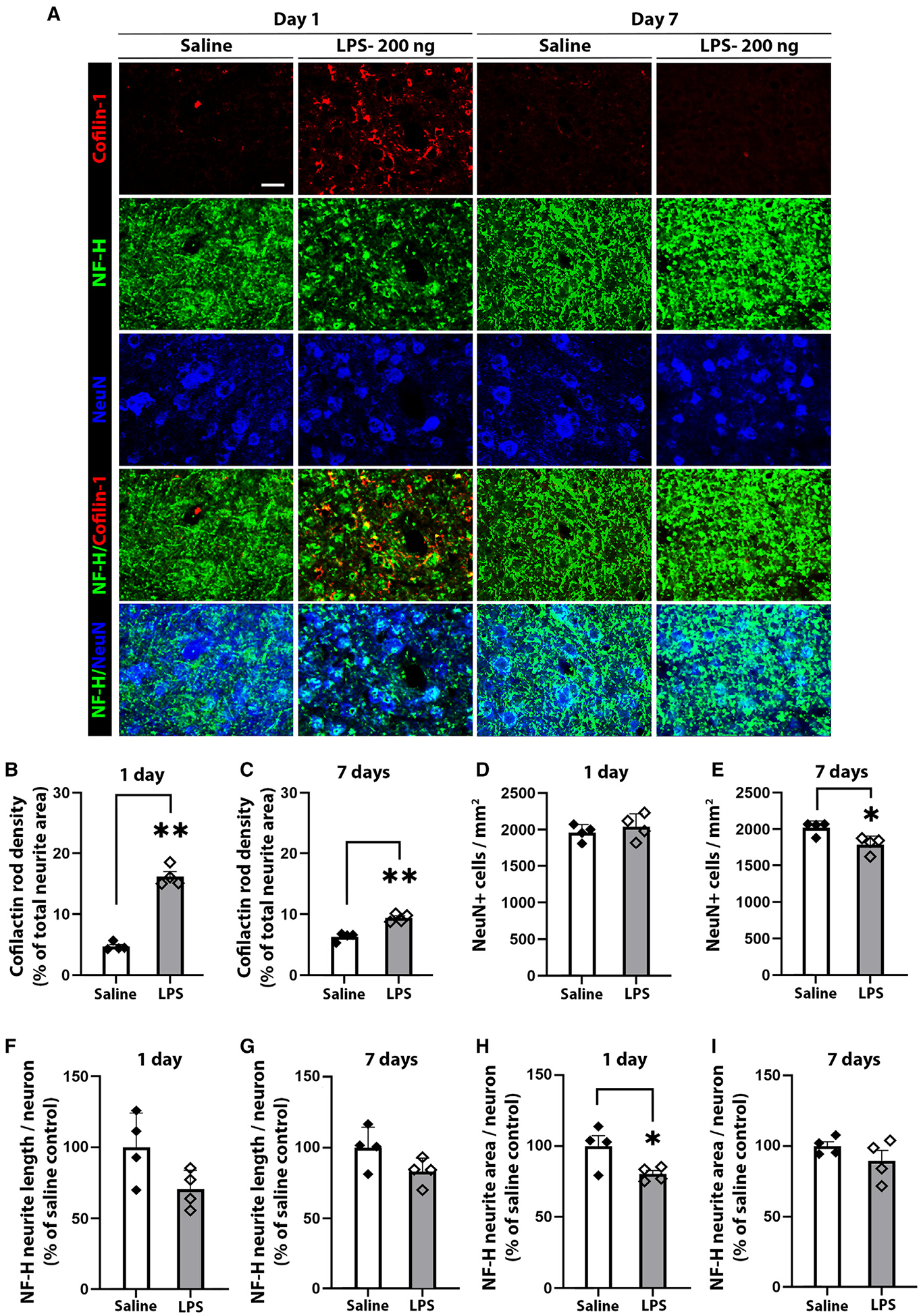
Lipopolysaccharide causes CAR formation and neurite loss (A) Photomicrographs from peri-injection cortex in WT mice at days 1 and 7 after injection of LPS (200 ng) or saline vehicle. CARs are identified by cofilin-1 (red), neurites by NF-H (green), and neuronal soma by NeuN (blue). Scale bar: 20 μm. (B and C) CAR density expressed as percentage of total neurite area. (D and E) Neuronal cell body density per mm^2^. (F and G) Neurite length per neuronal nucleus. (H and I) Neurite area per neuronal nucleus. Neurite length and area determinations are expressed relative to saline controls. n = 4; *p < 0.05 and **p < 0.01 by Student’s test. All data are shown as mean ± SEM.

To determine whether CAR formation with subsequent neurite loss is specific to S100β or is a more general consequence of the innate immune response, we also injected mouse cortices with LPS at a dose found to have minimal effect on neuronal cell body survival (Figure 5). The LPS injections likewise induced CAR formation and neurite loss, though the magnitude of these effects was less than observed with S100β.

### Roles of microglial NOX2 and neuronal cofilin-1 in inflammation-induced neuronal loss

We used mixed glial/neuronal cell culture preparations to characterize the interactions between these cell types that may drive inflammation-induced neurite loss. The cultures were comprised of WT or *COF*^−/+^ neurons plated onto a glial layer (containing astrocytes and microglia) derived from either WT or *p47*^phox−/−^ mice (Figure S6). In cultures containing WT neurons and WT glia, S100β produced a dose- and time-dependent induction of CARs (Figure S7). The CARs were restricted to the neurites and often caused an abrupt discontinuity of the microtubular cytoskeleton, as identified by immunostaining for microtubule-associated protein-2. The CAR formation was followed by a similar dose-dependent loss of neurites in these cultures (Figure S8). This neurite loss was not attributable to neuronal death, as inferred by propidium iodide exclusion (Figure S6), thereby indicating a preferential loss of the neuronal processes.

**Figure 6.**
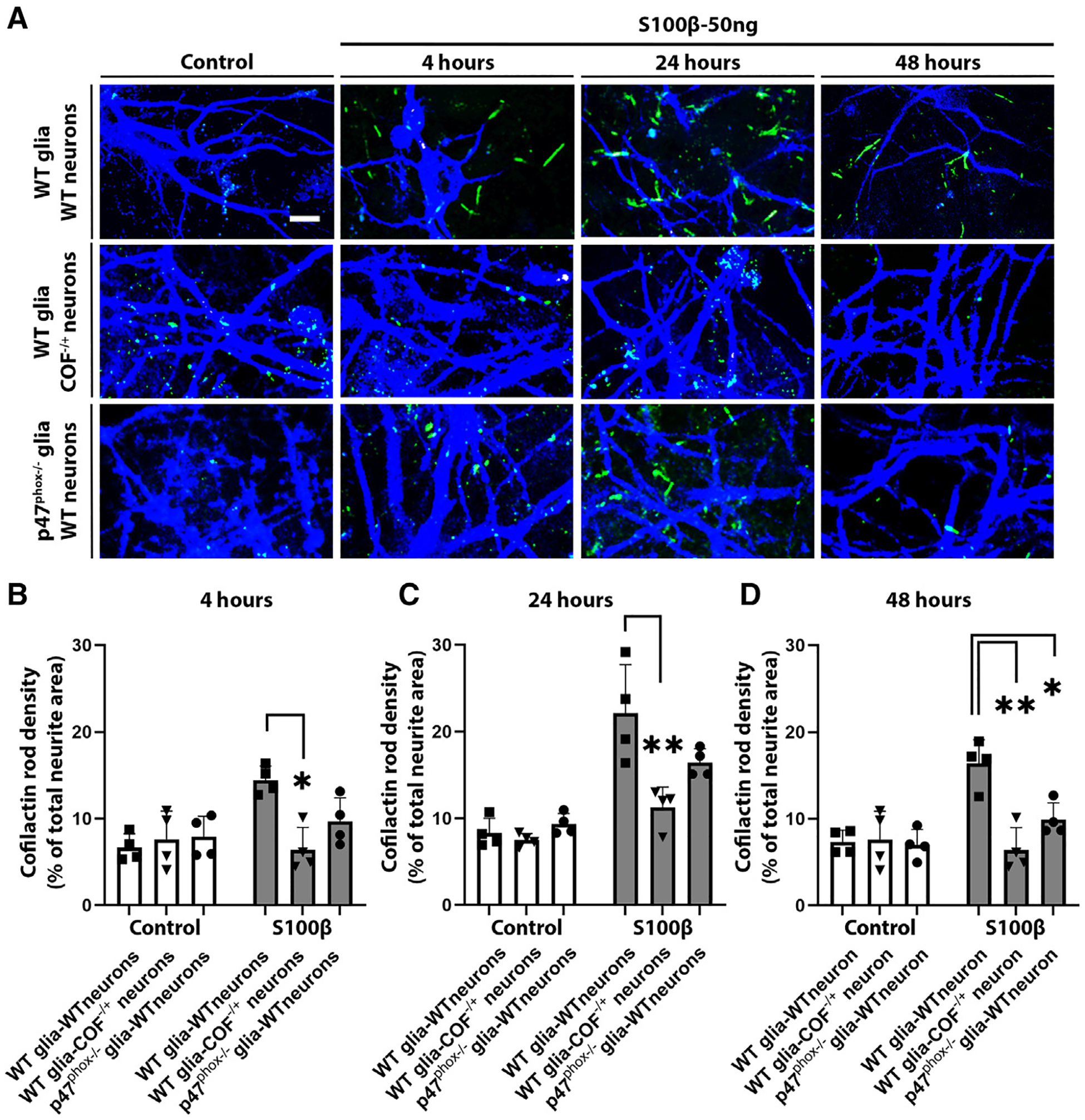
CAR formation in neuron-glia co-cultures is attenuated by both neuronal cofilin-1 hemizygosity and glial p47^phox^ deficiency (A) Representative photomicrographs of co-cultures immunostained for microtubule-associated protein-2 (MAP-2) (blue) and CARs (green). Scale bar: 10 μm. (B–D) CAR density expressed as percentage of total neurite area. n = 4; *p < 0.05 and **p < 0.01 vs. control by one-way ANOVA with Dunnett’s test. All data are shown as mean ± SEM.

**Figure 7.**
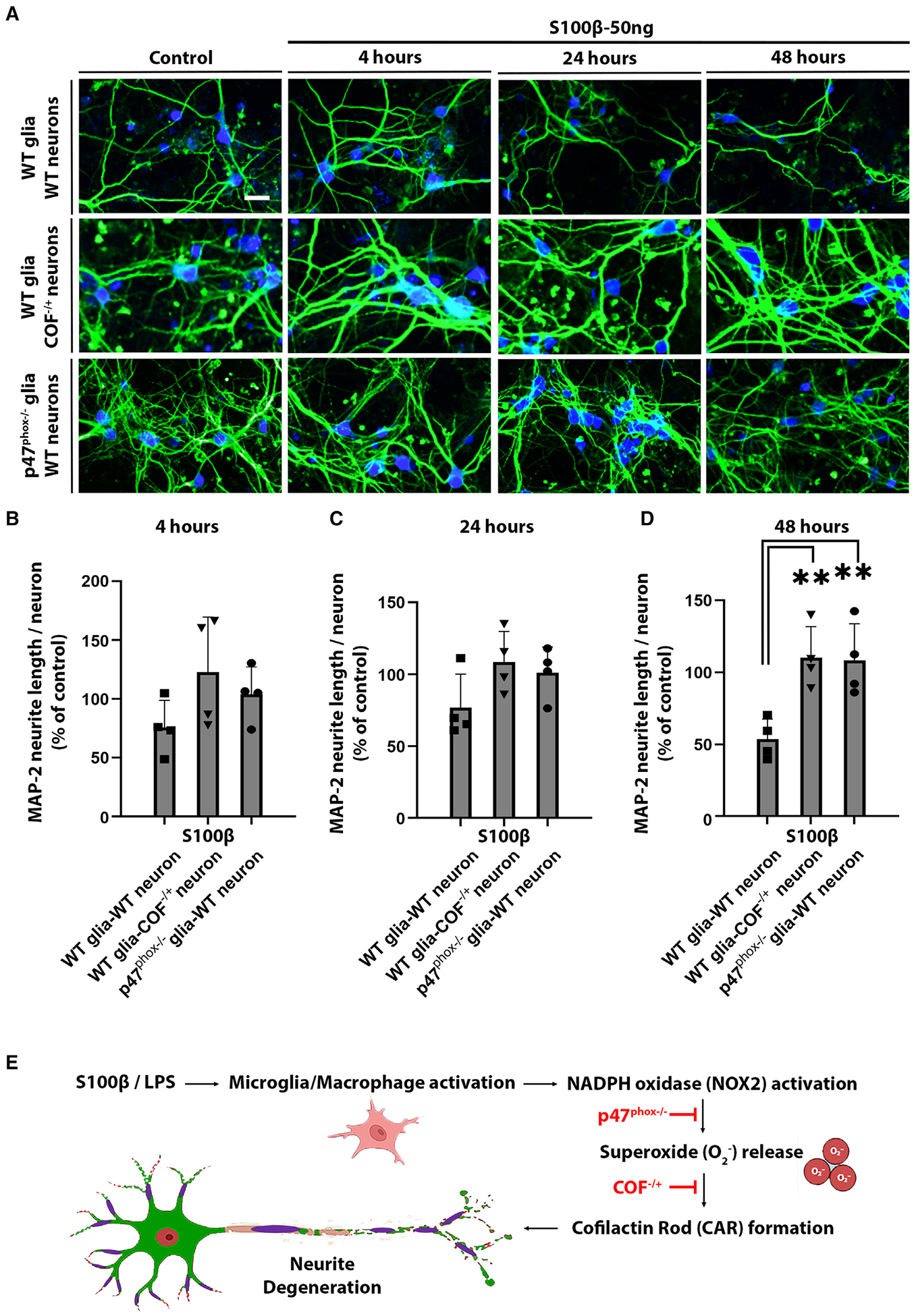
Neurite loss in neuron-glia co-cultures is attenuated by neuronal cofilin-1 hemizygosity and glial p47^phox^ deficiency (A) Photomicrographs of co-cultures immunostained for MAP-2 (green) and NeuN (blue). Scale bar: 20 μm. (B–D) Neurite length assessed at 4, 24, and 48 h of 50 ng/mL S100β incubation, expressed relative to control wells of the respective co-culture type. n = 4; *p < 0.05 and **p < 0.01 by one-way ANOVA with Dunnett’s test. (E) Mechanism proposed for inflammation-induced neurite degeneration. In response to pro-inflammatory stimuli, brain microglia and infiltrating macrophages upregulate superoxide production by NADPH oxidase. Resulting oxidative stress in nearby neurites leads to formation of CARs. Persistence of the CARs causes neurite degeneration, which can occur in the absence of parental neuron death. This process is attenuated in *p47*^phox−/−^ mice, which cannot form an active NADPH oxidase-2 complex, and in cofilin hemizygous (*COF*^−/+^) mice, which have reduced propensity to form CARs. All data are shown as mean ± SEM.

To confirm that the reduced CAR formation and neurite loss observed in *COF*^−/+^ mouse brain was attributable to decreased expression of cofilin-1 specifically in neurons, we evaluated S100β-induced CAR formation in cultures containing *COF*^−/+^ neurons and WT glia. These studies showed markedly reduced CAR formation at each of the time points evaluated, 4, 24, and 48 h (Figure 6), indicating a requisite role for neuronal cofilin-1 in the neurite loss. We then used cultures containing WT neurons plated onto *p47*^phox−/−^ glia to confirm that neurite loss was dependent upon glial (rather than neuronal) NOX2 activation. As expected, CAR formation in these cultures was markedly reduced relative to neurons plated onto WT glia at all time points examined (Figure 6). The reduced CAR formation in cultures containing *COF*^−/+^ neurons or *p47*^phox−/−^ glia was accompanied by a parallel reduction in neurite loss (Figures 7A–7D and S9)

## DISCUSSION

Pro-inflammatory M/M activation contributes to neurological disorders ranging from brain trauma to Alzheimer’s disease, but its effects on neuronal axons and dendrites are poorly characterized. The present studies show that axons and dendrites are particularly vulnerable to inflammatory injury. These studies also identify a mechanism by which inflammation-induced neurite injury occurs. Stimuli such as S100β released by damaged cells or LPS from bacterial membranes induce pro-inflammatory M/M responses, which include NOX2-mediated superoxide production and recruitment of circulating or perivascular macrophages to the area of tissue damage.^35–38^ The resulting oxidative stress on nearby neurites leads to CAR formation, which in turn leads to neurite degeneration and motor dysfunction (Figure 7E).

Prior evidence that CAR formation leads to neurite loss comes most definitively from studies using forced overexpression of cofilin-1, as cofilin-1 overexpression promotes CAR formation in the absence of other recognized cytotoxic processes.^17^ In primary neuron cultures, cofilin-1 overexpression causes CAR formation and subsequent neurite degeneration, likely by occluding normal anterograde and retrograde trafficking.^15,17,30,39^ Our findings further support a causal role for CARs in neurite loss by showing that inflammation-induced neurite loss is markedly reduced in neurons haploinsufficient in cofilin-1, both in cell culture and *in vivo*.

Our observations that CAR formation was also blocked in mice deficient in p47^phox^, an essential component of the activated NOX2 complex, confirms a role for superoxide production and oxidative stress in CAR formation and subsequent neurite degeneration. CAR formation requires both dephosphorylation of its serine-3 residue and the formation of cofilin dimers through intermolecular disulfide bonds,^13–15^ both of which are promoted by oxidative stress. Serine-3 is dephosphorylated by the slingshot phosphatase SSH-1L,^40^ which is normally sequestered to the scaffolding protein 14-3-3 but is released upon oxidation of 14-3-3ζ.^22^ Oxidative stress also promotes the intermolecular disulfide bonding between the cofilin-1 cysteines 39 and 147 that is thought to stabilize CARs.^11,14^ Our additional observation that S100β-induced CAR formation and neurite loss were largely prevented in the co-cultures containing glia from *p47*^phox−/−^ mice argues against a role for neuronal NOX2 in these processes; however, neuronal NOX2 activation has been identified as a requisite step in CAR formation induced by a cellular prion protein-dependent pathway.^41^

Neurite loss not attributable to parent neuron death has been identified in brain ischemia^42,43^ brain trauma,^44,45^ neurodegenerative disorders,^18,33^ and sepsis.^46,47^ Here, the observations that inflammation-induced neurite loss occurred independently of parent neuronal cell demise was confirmed using both YFP and immunolabeled cells and in both cell cultures and mouse brain. Most of these studies used S100β and LPS at concentrations titrated to induce neurite loss in the absence of neuronal cell body loss. We considered the possibility that the neurite loss observed 1–3 days after S100β injection might simply be a precursor or sign of incipient neuronal death but found no detectable loss of neurons or reduction in neuronal nuclear size at time points up to 14 days after the injections.

We chose S100β as the inflammatory stimulus because it is an endogenous alarmin that is released by injured cells in conditions such a stroke and trauma and is thought to be a major driver of the innate immune response in those conditions.^23,27,28^ The similar results obtained using LPS indicate that the greater vulnerability of neurites to inflammatory injury is not unique to the S100β stimulus. The larger effects observed with S100β are consistent with the relative effects of S100β and LPS on microglial activation *in vitro*.^28^

Mouse motor dysfunction was evaluated using the corner test, which has been widely adopted for this purpose.^34^ WT mice with S100β injected into the motor cortex showed significant impairment, while the *COF*^−/+^ or *p47*^phox−/−^ mice did not, in agreement with the histological measures of neurite loss. Recovery with time after the injections is difficult to interpret given the multiple processes known to contribute to recovery after injury to the motor cortex. Nevertheless, the motor impairment induced by inflammation in the absence of neuronal cell loss highlights the relevance of cortical neurite loss to functional impairment after stroke and other conditions in which the innate immune response is activated.

The teleological role of CAR formation in mature neurites remains uncertain, but evidence suggests that, over short time intervals, this conserves local ATP levels by blocking actin-dependent processes.^15,16,18^ However, CAR formation that is extensive or sustained leads to neurite degeneration,^7–10^ as shown here. We additionally show that the brain inflammatory response is sufficient to induce this mechanism of neurite degeneration and that interventions targeting either NOX2 activation or CAR formation can be effective in limiting inflammation-induced neurite degeneration.

### Limitations of the study

Our results used an artificial stimulus that produced inflammation in the absence of primary neuronal injury to prove the principle that neurites are particularly vulnerable to inflammation-induced degeneration. However, stimuli such as stroke and brain trauma induce inflammatory responses over a range of intensities that depend upon distance from the primary injury site, the presence or absence of blood products, and other factors. This spectrum of inflammatory responses would thus be expected to cause areas of neuronal death in addition to areas of more selective neurite loss. Both neuronal cell loss and selective neurite loss can cause functional impairment, and the relative contribution of these processes to net functional impairment after injuries such as stroke or trauma remains to be determined.

We used *p47*^phox−/−^ mice and cells cultured from these mice to assess the role of superoxide production in CAR formation and neurite loss, as NOX2 requires p47^phox^ to form an active NOX2 complex. A limitation of this approach is that p47^phox^ deficiency could have other effects that influence M/M response to inflammatory stimuli. Studies using pharmacological NOX2 inhibition would obviate this concern and at the same time test a potential therapeutic approach.

Our cell culture studies show that activation of NOX2 in cells other than neurons is sufficient for S100β-induced CAR formation but do not establish whether this is microglial or astrocyte NOX2. M/M express NOX2 at far higher levels than astrocytes,^48,49^ but all three cell types may contribute to the process of CAR formation and neurite injury. Similarly, microglia and infiltrating macrophages contribute to the innate brain response *in vivo* but are not distinguished from one another by the Iba-1 labeling used here.^3,38^ We thus cannot estimate the relative contributions of these different cell types to CAR formation and neurite loss.

## STAR★METHODS

### RESOURCE AVAILABILITY

#### Lead contact

Further information and requests for resources and reagents should be directed to and will be fulfilled by the lead contact, Raymond Swanson (raymond.swanson@ucsf.edu).

#### Materials availability

This study reports a novel mouse line, cofilin hemizygous (COF^−/+^) mice. These mice will be made available upon request to the lead author.

#### Data and code availability

- All aggregated data are available in the main text and figures or the supplementary materials. Primary data (e.g., images and spreadsheets) will be provided upon written request to the lead contact. The primary data have also been deposited into the Mendeley data repository at https://data.mendeley.com/datasets/829ywfmvrn/1.
- This paper does not report any original code.
- Any additional information can be requested directly from the lead contact.

### EXPERIMENTAL MODEL AND SUBJECT DETAILS

#### Mice

Studies were approved by the Institutional Animal Care and Use Committees at the San Francisco Veterans Affairs Medical Center and Colorado State University and were performed in accordance with the National Institutes of Health Guide for the Care and Use of Laboratory Animals. Results are reported in accordance with the ARRIVE guidelines. C57Bl/6 wild-type, Thy1-YFP, and p47^phox−/−^ mice were obtained from Jackson Laboratories. The p47^phox−/−^ colony has been maintained as homozygotes and backcrossed to C57Bl/6 wild-type mice every 10 generations. Cofilin hemizygous (COF^−/+^) mice were generated as described in Figure S4, and subsequently back-crossed >8 generations to C57Bl/6 mice.

#### Cell culture preparations

Glial cultures, containing astrocytes and microglia, were prepared from postnatal day 0 or 1 C57BL/6 WT and p47^phox−/−^ mice of both sexes as described.^50^ The dissociated cells were plated and maintained in a 37°C, 5% CO_2_ incubator in DMEM (ThermoFisher) containing 10% fetal bovine serum (FBS), 5 mM glucose, 1 mM pyruvate, 2 mM L-glutamine, 100U/ml penicillin, and 100 μg/mL streptomycin. At confluency, the cells were lifted by trypsin-EDTA and re-plated onto poly-D-lysine - coated coverslips at a density of 2 × 10^5^ cells/12 mm coverslip. Neurons from WT or cofilin-1 hemizygous (COF^−/+^) mice were isolated from embryonic day 16 mice as described^51^ and plated directly onto the glial cultures. The co-cultures were then maintained in Neurobasal A medium (Thermo-Fisher, #21103049), containing 5 mM glucose, 0.2 mM pyruvate 1 mM Glutamax (ThermoFisher), and B27 supplement (ThermoFisher) in a 5% CO_2_, 37°C incubator.

### METHOD DETAILS

#### Western blotting and characterization of cofilin-1 hemizygous mice

The hindbrains of embryonic (E10.5) mouse pups were quickly frozen in liquid nitrogen. Protein was solubilized by probe sonication into 100 μL Laemmli sample buffer, boiled for 3 min, run on a 15% SDS-PAGE gel, then transferred to nitrocellulose. Blots were blocked with bovine serum albumin, then incubated with previously characterized primary antibodies, detected on an Odyssey Infrared scanner and quantified with normalization to GAPDH. Two dimensional western blots were performed as described.^21,52^ After transfer to nitrocellulose and blocking, cofilin and its phosphorylated form were immunolabelled with rabbit pan ADF/cofilin antibody.^21,53^

#### Intracortical injections

All studies used equal numbers of male and female mice, age 3–4 months. Stereotaxic intracortical injections of lipopolysaccharide (LPS; Millipore), recombinant mouse S100β (Novus Biologicals) or saline (vehicle) were performed under 1.5% isoflurane anesthesia. Injections were made into the right primary motor cortex (AP 1.0, ML 2.0, DV 1.0 mm from bregma) in 0.5 μL volumes at 0.2 μL/minute. In some studies, the S100β was denatured by heating to 95°C for 60 min.

#### Motor function testing

Mice were placed at the center of the corner apparatus consisting of two adjacent walls that form a 30-degree angle.^54^ The direction the mouse turned when it reached the corner was recorded over 10 trials on each day of testing. Data are presented as the percentage of turns made to the left.

#### Histology

Anesthetized mice were perfused with ice-cold saline followed by a solution of 4% paraformaldehyde in phosphate-buffered saline (PFA). Brains were removed and post-fixed in 4% PFA for 24 h, then immersed for another 24 h in 20% sucrose for cryoprotection, frozen, and cut into 40 μm coronal sections with a cryostat. The sections were permeabilized with 95% methanol and 5% 0.1 M phosphate buffer (PB) at −20°C for 15 min, then incubated with 10 mM sodium citrate (pH: 6.0) at 80°C for 30 min. The sections were incubated with unconjugated donkey anti-mouse IgG Fab fragments (Jackson Immunoresearch), diluted 1:35 in 0.1 M PB overnight at 4°C. On the following day the sections were incubated in blocking buffer (2% donkey serum and 0.1% bovine serum albumin (BSA) in 0.1 M PB) for 1 h, and then incubated with primary antibody overnight at 4°C. After washing, the sections were incubated with secondary antibodies for 1 h or, where labeling was additionally performed with biotin-tagged primary antibodies, the sections were incubated with fluorescently tagged streptavidin (Jackson Immunoresearch, 1:250 in 0.1 M PB). Stained sections were mounted on glass slides in antifade mounting medium (Vector laboratories). Control sections omitting either primary or secondary antibodies showed no signal above background.

Primary antibodies were obtained from the following sources and used at 1:500 dilutions: rabbit anti-cofilin-1, Cytoskeleton #ACFL02, Denver, CO; mouse anti-MAP-2, Millipore #3418, Temecula, CA; mouse anti-NeuN, Millipore #MAB377B, Temecula, CA; mouse anti-NF-H, BioLegend #SMI31, San Diego, CA; rabbit anti-Phospho-Histone H2AX (Ser139) Antibody #2577; rabbit Iba1, Wako 019–19741. Secondary antibodies were obtained from the following sources and used at designated dilutions: Alexa Fluor 488 Donkey Anti-Mouse IgG (H + L) (1:500, Invitrogen # A-21202), Alexa Fluor 594 Donkey Anti-Rabbit IgG (H + L) (1:500, Invitrogen #A-11058), Alexa Fluor 405 Goat Anti-Mouse IgG (H + L) (1:250, Invitrogen # A-31553), Alexa Fluor 488 Goat Anti-Rabbit IgG (H + L) (1:500, Invitrogen # A-11008), DyLight 405 Streptavidin (1:250, Jackson Immunoresearch #016-470-084).

#### Cell culture studies

Studies were initiated by exchanging medium to fresh medium omitting the B27 supplement. Recombinant mouse S100β or LPS were added from concentrated stocks. Immunostaining of cell cultures was performed as described for brain sections but without the incubations in sodium citrate or donkey anti-mouse IgG Fab fragments. Where used to assess cell viability, propidium Iodide was added to the cultures 10 min before fixation.

#### Image analysis

All photographs were prepared with the same microscope under identical illumination and data capture conditions. Both the persons taking the photographs and analyzing the immunostaining were blinded to the treatment conditions. For cell culture studies, images were taken from five arbitrary regions of each cell culture well. For brain sections, confocal images were taken at four pre-determined locations 0.5 mm to the injection site on two sections from each animal. The confocal images were z-stacked, and each processed image was prepared using a stack of 10 1-μm thick Z-stage images. For assessments of CAR formation, images were thresholded using the Triangle function in ImageJ. The area of CAR signal was measured and normalized in two ways. First, it was expressed as percent of the NF-H area plus cofilactin area (for WT mice) or MAP-2 area plus cofilactin area (for cell cultures), because MAP2 and NF-H signals are lost where CARs form. As a second approach, the CAR area measurements were normalized to neuronal number. In each case, the CAR values obtained in each brain section image were averaged to provide an aggregate value for each brain, and values in the culture wells were averaged to provide an aggregate value for each independent cell culture experiment. Neurite area measurements were made after thresholding the NF-H or MAP-2 images using the ImageJ Otsu or Li functions, respectively. Total YFP-expressing or NF-H-stained neurite length for each image was quantified by summing the length of neurites after skeletonizing the images in ImageJ. The neurite area and neurite length measurements in each image were normalized to the number of neuronal cell bodies in the image, as assessed by YFP (+) soma or NeuN immunostaining.

Microglia/macrophage responses to S100β or LPS were assessed by 3 methods.^37^ The total number of Iba1+ cells was assessed using ImageJ/Fiji macro. Activated Iba1+ cells were manually labeled based on the previously published parameters.^37^ Iba1+ microglial extension area was quantified after excluding the cell body areas on ImageJ using the particle size function. γH2AX staining intensity was measured in neuronal nuclei of cell cultures as defined by NeuN. Neurons were designated as “γH2AX-positive” when the integrated nuclear γH2AX density exceeded the 80^th^ percentile of values measured in the corresponding control images for that experiment. Thus, by definition, 20% of neurons were γH2AX-positive in all control wells.

### QUANTIFICATION AND STATISTICAL ANALYSIS

The “n” of each study defined as the number of mice or, for cell cultures, the number of independent experiments. Each independent experiment contained triplicate or quadruplicate culture wells. Numerical data were expressed as means ± SEM and analyzed using one-way ANOVA by either the Tukey–Kramer test where multiple groups were compared against one another, or Dunnett’s test where multiple groups were compared against a common control group. Where only two groups were compared, the two-sided t test was used. All data analysis was performed by individuals who were blinded to the experimental conditions.

## Supplementary Material

1

## Figures and Tables

**Table:** KEY RESOURCES TABLE

REAGENT or RESOURCE		SOURCE	IDENTIFIER
Antibodies			
Affinity purified rabbit anti-cofilin-1		Cell Signaling Technologies	Cat# 51755, RRID: AB_10622000
Rabbit pan ADF/cofilin antibody #1439		Prof. James Bamburg, Colorado State University	N/A
Mouse anti-Cofilin-1 antibody mAb22		Prof. James Bamburg, Colorado State University	N/A
Mouse anti-Neurofilament H (NF-H/SMI31)		Biolegend	Cat# 801601, RRID: AB_2564641
Mouse anti-Microtubule associated protein (MAP-2)		Millipore-Sigma	Cat# MAB3418, RRID: AB_11212326
Biotin tagged Mouse anti-Neuronal Nuclei (NeuN)		Millipore-Sigma	Cat# MAB377B, RRID: AB_177621
Rabbit Anti-Iba1		Wako	Cat# 019-19741, RRID: AB_839504
Mouse anti-GAPDH		Millipore-Sigma	Cat# mAb374, RRID: AB_2107445
Streptavidin, DyLight 405		Jackson Immunoresearch	Cat# 016-470-084, RRID: AB_2337248
Donkey Anti-Mouse, Alexa Fluor 488		Life technologies	Cat# A21202, RRID: AB_141607
Donkey Anti-Rabbit, Alexa Fluor 594		Invitrogen	Cat# A21207, RRID: AB_141637
Donkey anti-mouse IgG Fab fragments		Jackson Immunoresearch	Cat# 715-007-003, RRID: AB_2307338
Goat Anti-Mouse, DyLight 680		ThermoFisher	Cat# PI35518, RRID: AB_614942
Goat Anti-Rabbit, DyLight 800		ThermoFisher	Cat# PISA510036, RRID: AB_2556774
Chemicals, peptides, and recombinant proteins			
DMEM, no glucose, no glutamine, no phenol red		ThermoFisher	Cat# A1443001
Fetal Bovine Serum (FBS)		ThermoFisher	Cat# A5670501
Glucose		ThermoFisher	Cat# A2494001
Pyruvate		ThermoFisher	Cat# 11360070
L-glutamine		ThermoFisher	Cat# A2916801
Penicillin- Streptomycin		ThermoFisher	Cat# 15070063
Trypsin-EDTA		ThermoFisher	Cat# 25200072
Poly-D-lysine		ThermoFisher	Cat# A3890401
Neurobasal^™^-A Medium, no D-glucose, no sodium pyruvate		ThermoFisher	Cat# A2477501
Glutamax		ThermoFisher	Cat# 35050061
B27 supplement		ThermoFisher	Cat# 0080085SA
Recombinant mouse S100β		Novus Biologicals	Cat# NBP2-53070
Lipopolysaccharide (LPS)		Millipore-Sigma	Cat# L2630
Propidium Iodide		ThermoFisher	Cat# P1304MP
Nitrocellulose membrane		ThermoFisher	Cat# 45-004-010
Experimental models: Organisms/strains			
C57Bl/6 wild-type (WT) mice		Jackson Laboratories	Strain: #:000664 RRID: IMSR_JAX:000664
Thy1-YFP mice		Jackson Laboratories	Strain #:003709 RRID: IMSR_JAX:003709
Cofilin hemizygous (COF^−/+^) mice		Prof. James Bamburg, Colorado State University	N/A
p47^phox−/−^ mice		Jackson Laboratories	Strain #:027331 RRID: IMSR_JAX:027331
Software and algorithms			
ImageJ/Fiji	NIH		https://imagej.net/software/fiji/
Prism- GraphPad	Dotmatics		https://www.graphpad.com/scientific-software/prism/www.graphpad.com/scientific-software/prism/
Endnote 20	Clarivate		https://endnote.com/?language=en
Adobe Photoshop	Adobe		https://www.adobe.com/products/photoshop.html/
Adobe Illustrator	Adobe		https://www.adobe.com/products/illustrator.html/

## Highlights

- The brain inflammatory response causes neurite (axon and dendrite) degeneration
- Neurites are more vulnerable than neuronal cell bodies to inflammatory injury
- Inflammatory neurite loss results from the formation of cofilactin rods
- Inflammatory neurite loss is associated with functional impairment
