# Supplementary material for: Cofilactin rod formation mediates inflammation-induced neurite degeneration
Source: Cell Rep. Author manuscript; Available in PMC 2024 May 3. (PMC11068216; doi:10.1016/j.celrep.2024.113914)
Supplement: 1 [file NIHMS1988677-supplement-1.pdf]

Cell Reports, Volume *43*

Supplemental information

Cofilactin rod formation mediates inflammation-induced neurite degeneration

Gökhan Uruk, Ebony Mocanu, Alisa E. Shaw, James R. Bamburg, and Raymond A. Swanson

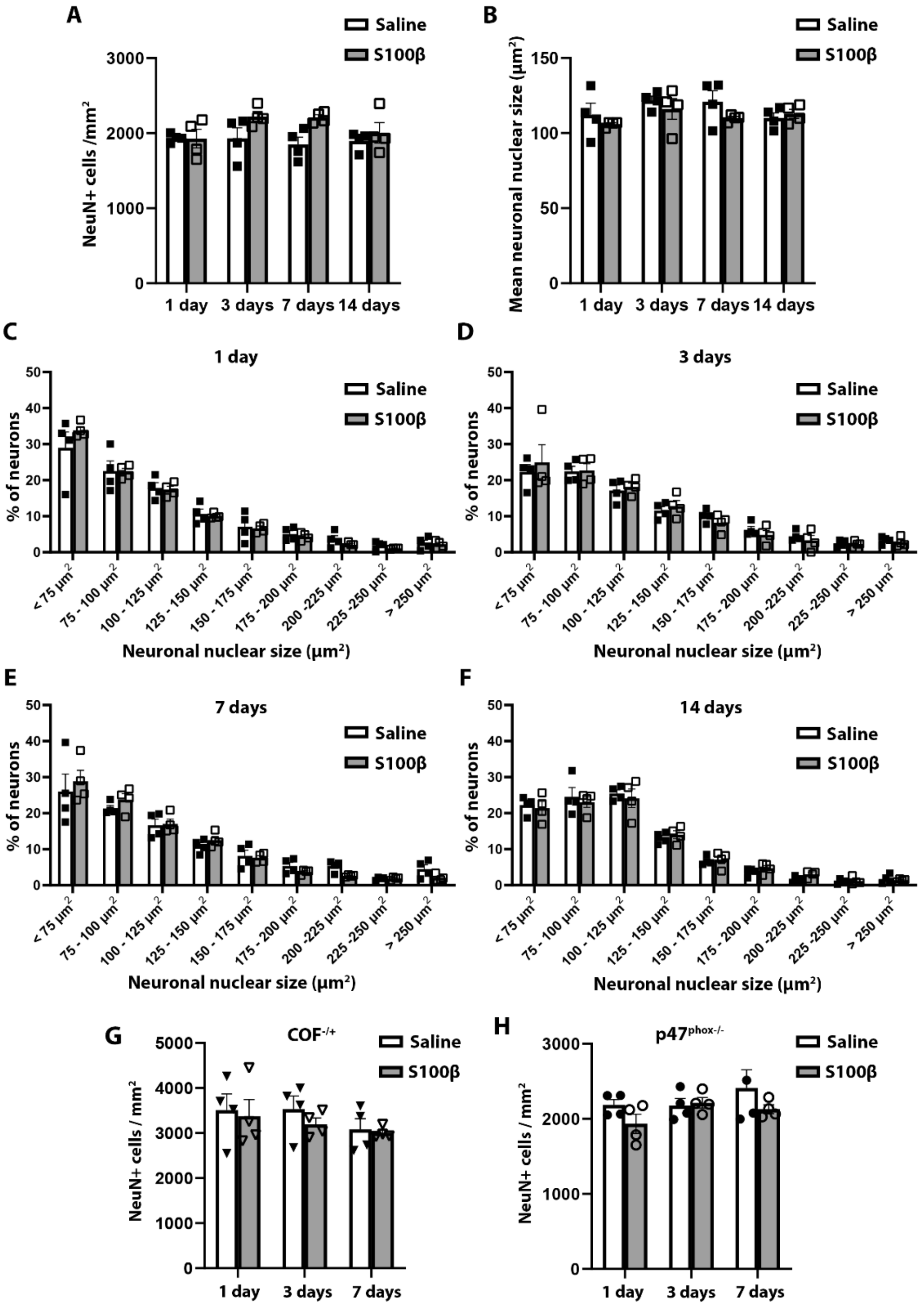

Figure S1, related to Figures 2 and 3. Neuronal loss as assessed by neuronal density and nuclear size.

(A, B) Quantification of neuronal cell body density and neuronal nuclear size at 1, 3, 7, and 14 days after 50 ng S100β injections in WT mice. (**C-F**) Histograms showing distribution of neuronal nuclei by size. (**G-H**) Quantification of neuronal cell body density in $COF^{-/+}$ and $p47^{phox-/-}$ mice after 50 ng S100β injections. n = 4; differences between saline and S100β not significant by ANOVA with Tukey tests between each pair of data. All data are shown as mean ± SEM.

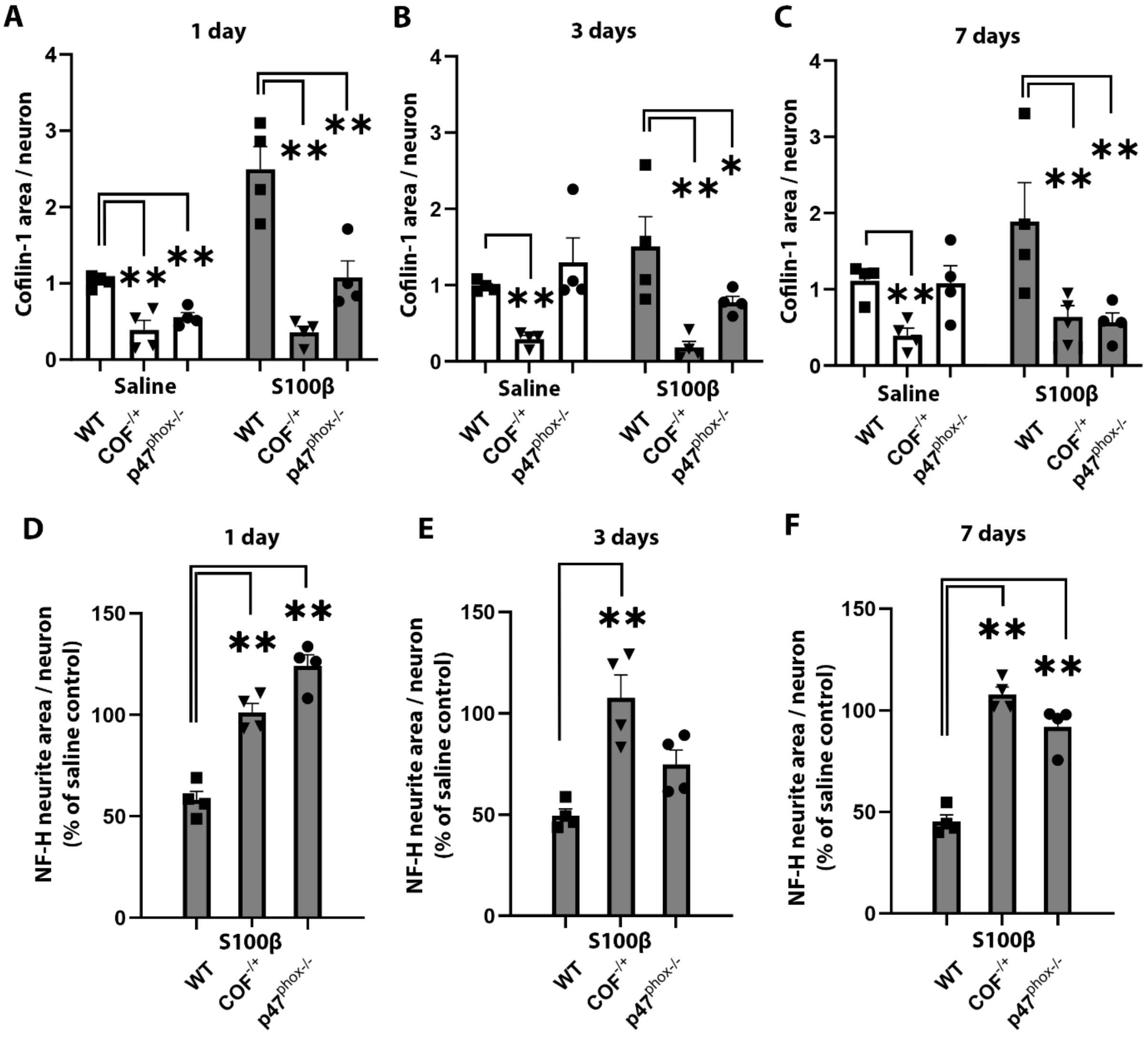

Figure S2, related to Figure 3. Cofilactin rod (CAR) formation and neurite loss as assessed by cofilactin and neurofilament-H area.

(A-C) Cofilactin rod area in mice of each genotype injected with saline or 50 ng S100β, normalized to neuron number. (Corresponding to the cofilactin rod / total neurite length assessments in Figure 3B-D). **(D-F)** NF-H+ neurite area in mice of each genotype injected with 50 ng S100β, normalized to neuron number and expressed relative to the corresponding saline-injected mice of each genotype (Corresponding to the neurite length assessments in Figure 3F-H). n = 4; *$p < 0.05$, **$p < 0.01$ by one-way ANOVA with Dunnett's test. All data are shown as mean ± SEM.

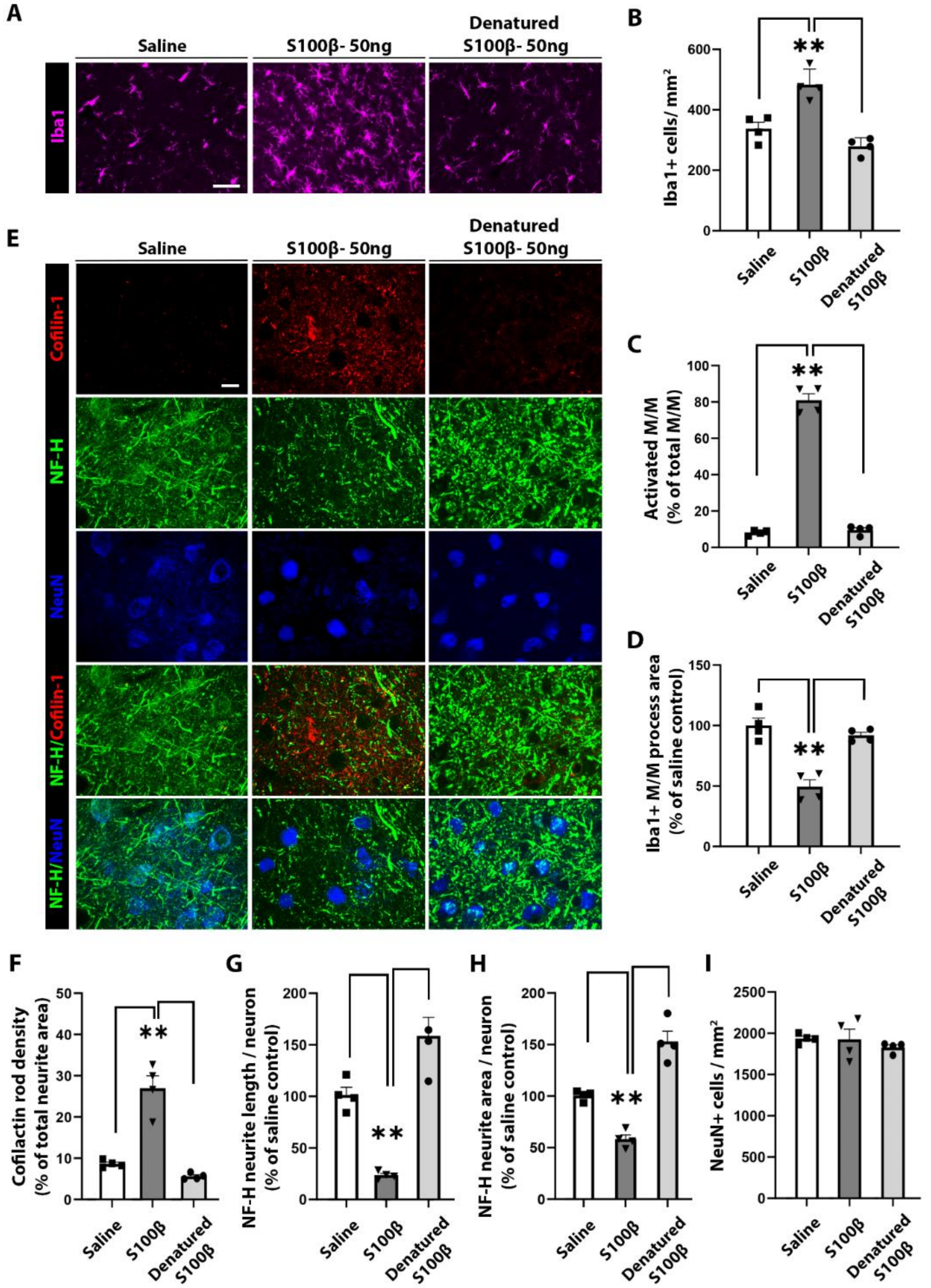

Figure S3, related to Figure 4. Denatured S100β does not induce microglia/macrophage activation, CAR formation, or neurite loss.
Photomicrographs from peri-injection cortex 1 day after injection of saline, 50ng S100β, or 50ng denatured S100β. **(A)** Microglia/macrophage (M/M) morphology by Iba1 immunostaining. Scale bar = 20 µm. **(B-D)** Quantification of M/M responses. **(E)** CAR formation identified by cofilin-1 (red) and neurites identified by neurofilament-H (NF-H), green), with neuronal nuclei identified by NeuN (blue). Scale bar = 20 µm. Graphs show quantification of CAR density **(G)**, NF-H length per neuronal nucleus **(H)**, NF-H-area per neuronal nucleus **(I)**, and neuronal cell density. The length and area determinations are expressed relative to saline controls prepared in parallel. n = 4; *p < 0.05, **p < 0.01 by one-way ANOVA with Dunnett's test. All data are shown as mean ± SEM.

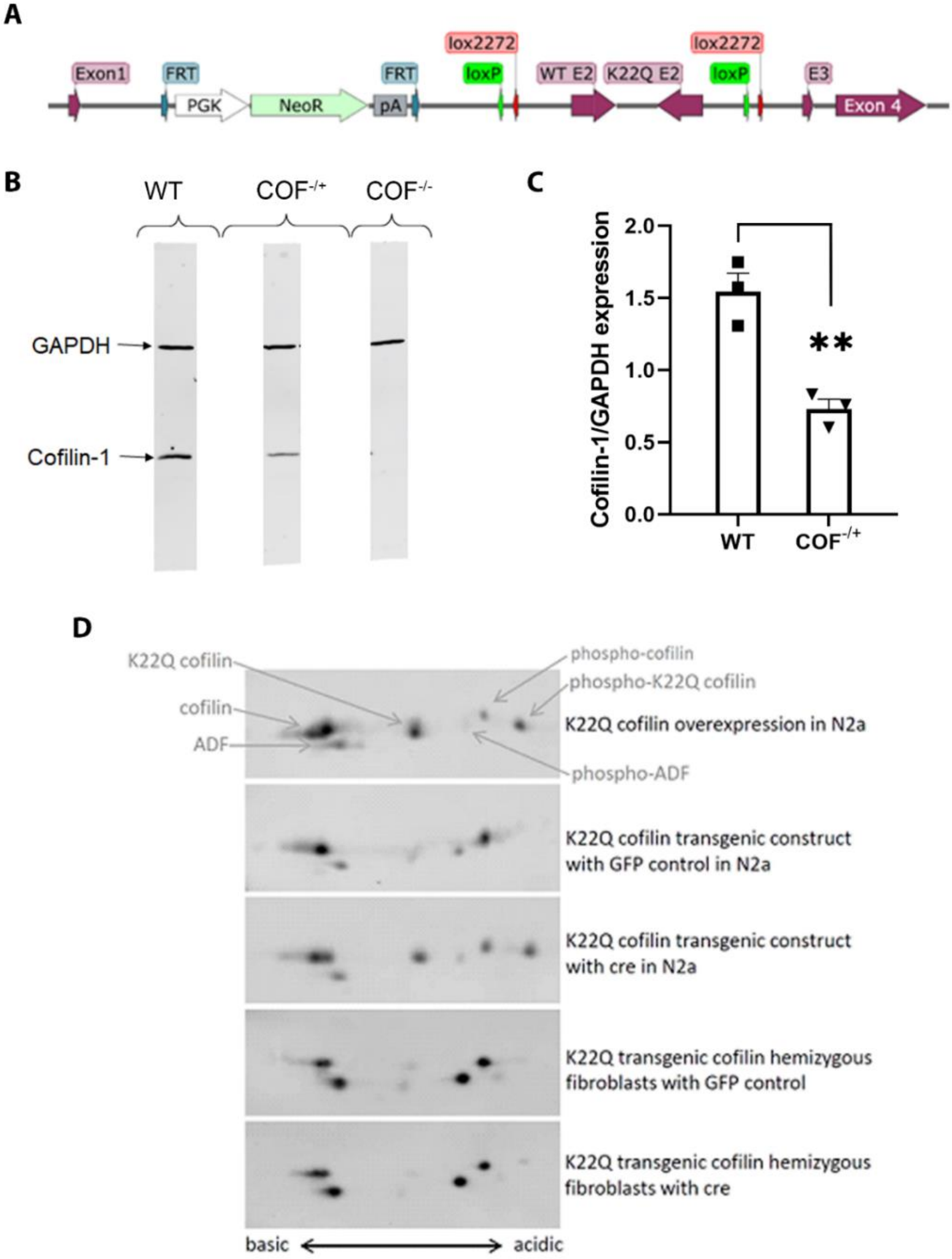
A
Exon1
FRT
PGK
NeoR
FRT
pA
lox2272
loxP
WT E2
K22Q E2
lox2272
loxP
E3
Exon 4
B
WT
COF-/+
COF-/-
GAPDH
Cofilin-1
C
Cofilin-1/GAPDH expression
2.0
1.5
1.0
0.5
0.0
**
WT
COF-/+
D
K22Q cofilin
phospho-cofilin
phospho-K22Q cofilin
cofilin
ADF
phospho-ADF
K22Q cofilin overexpression in N2a
K22Q cofilin transgenic construct with GFP control in N2a
K22Q cofilin transgenic construct with cre in N2a
K22Q transgenic cofilin hemizygous fibroblasts with GFP control
K22Q transgenic cofilin hemizygous fibroblasts with cre
basic
acidic

Figure S4, related to Figures 3 and 4. Generation of COF$^{-/+}$ mice.
The cofilin hemizygous mouse line used in this study was the result of an attempt to generate a conditional cofilin K22Q knock-in mouse that would not form cofilactin rods. The transgenic mouse was designed to have an inverted duplication of cofilin exon 2, in which wildtype cofilin would be expressed until irreversible inversion by cre recombinase to instead express a K22Q mutant cofilin that has reduced F-actin binding affinity and does not form CARs [S1]. The design of the transgenic targeting cassette is shown in panel **(A**) The presence of the transgenic allele in the resulting mouse line was confirmed by PCR and by DNA sequencing. Unexpectedly, the transgenic cofilin exon 2 was silent, expressing no wild-type or K22Q cofilin before or after cre inversion, resulting in a cofilin null allele. **(B, C)** Western blot gels and quantifications showed that the hemizygous K22Q cofilin mice express approximately half the wild-type amount of cofilin, and the homozygous mice are cofilin null. n = 4; *p < 0.05, **p < 0.01 by Student's t-test. Data are shown as mean ± SEM. **(D)** The lack of expression from the transgenic K22Q allele was further confirmed by expressing cre in hemizygous mouse dermal fibroblasts and detecting cofilin separated by 2D gel electrophoresis. Due to its altered p**I**, K22Q cofilin separates as distinct spots from wildtype cofilin during 2D gel electrophoresis, as shown by overexpression of K22Q cofilin in mouse N2a cell line, as well as by transfection of the K22Q cofilin transgenic targeting construct into N2a cells with or without cre co-expression. Primary dermal fibroblasts from hemizygous K22Q cofilin transgenic mouse pups were infected with adenovirus expressing either cre and GFP, or GFP alone.

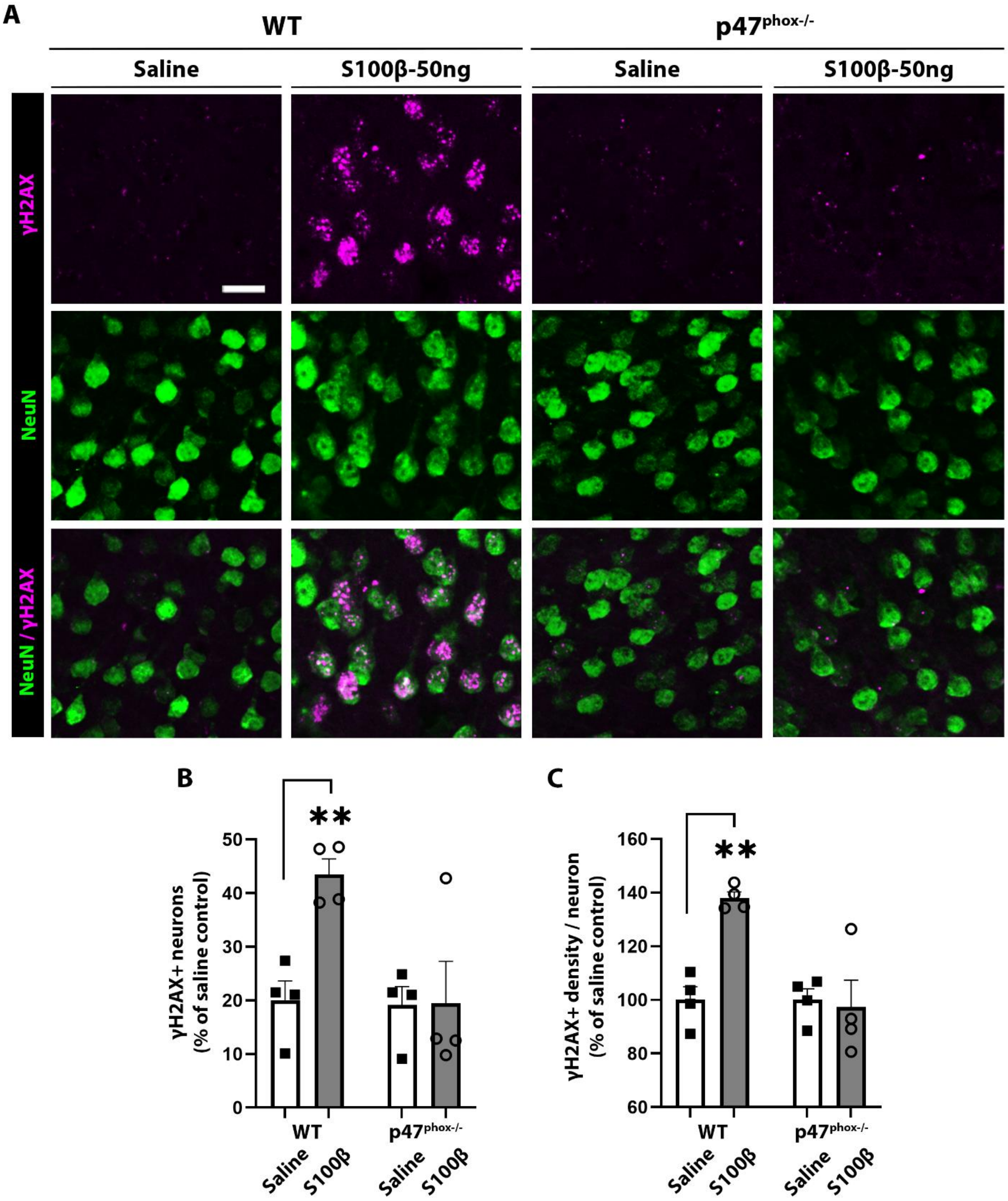

Figure S5, related to Figures 3 and 4. S100β induced neuronal oxidative stress.
(A) Foci of γH2AX (magenta), which form at sites of DNA strand breaks, in nuclei of neurons immunostained with NeuN (green) in WT, but not $p47^{phox-/-}$ mice. Scale bar = 20 µm. Data are quantified in **(B)** as percent of neurons with detectable γH2AX signal and in **(C)** as mean integrated γH2AX signal density per neuron. Data are expressed relative to saline-injected controls. **$p < 0.01$ by Student's t-test. All data are shown as mean ± SEM.

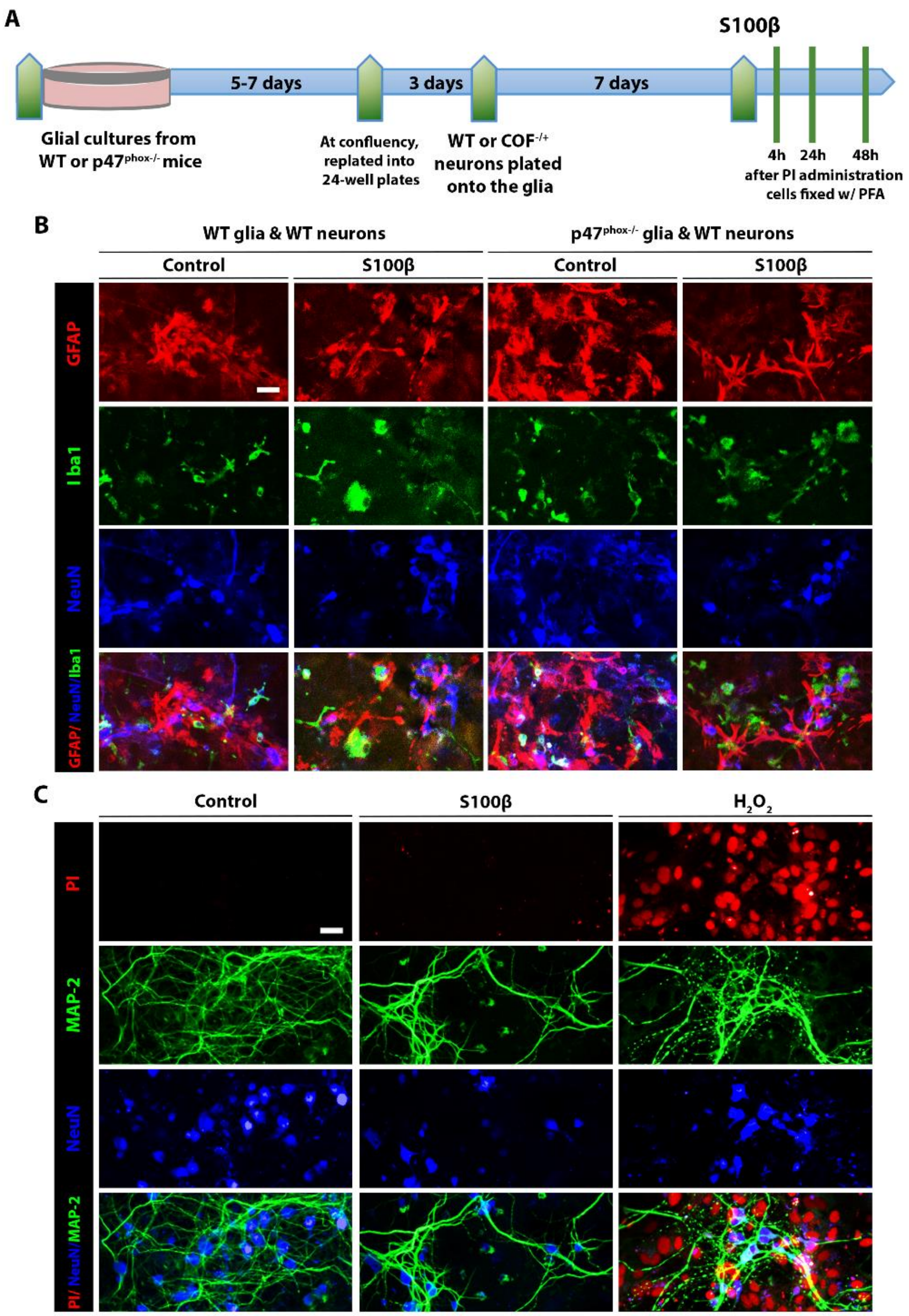

Figure S6, related to Figures 6 and 7. Characterization of cell culture preparations. **(A)** Schematic of co-culture preparations. **(B)** Immunolabeling for astrocytes (GFAP; red), microglia (Iba1; green) and neuronal somas (NeuN; blue) in co-cultures with or without 50 ng / ml S100β incubation for 48 hours. **(C)** Immunolabeling for neuronal somas and neurites with NeuN (blue) and microtubule-associated protein-2 (MAP-2; green) after propidium iodide (PI; red) incubation. 48 - hour incubations in 50 ng / ml S100β caused no detectable neuron death, as detected by PI permeability. H_2O_2 (10 μM) provides as a positive PI control. Scale bar = 20 μm. Representative of n = 3 co-culture preparations.

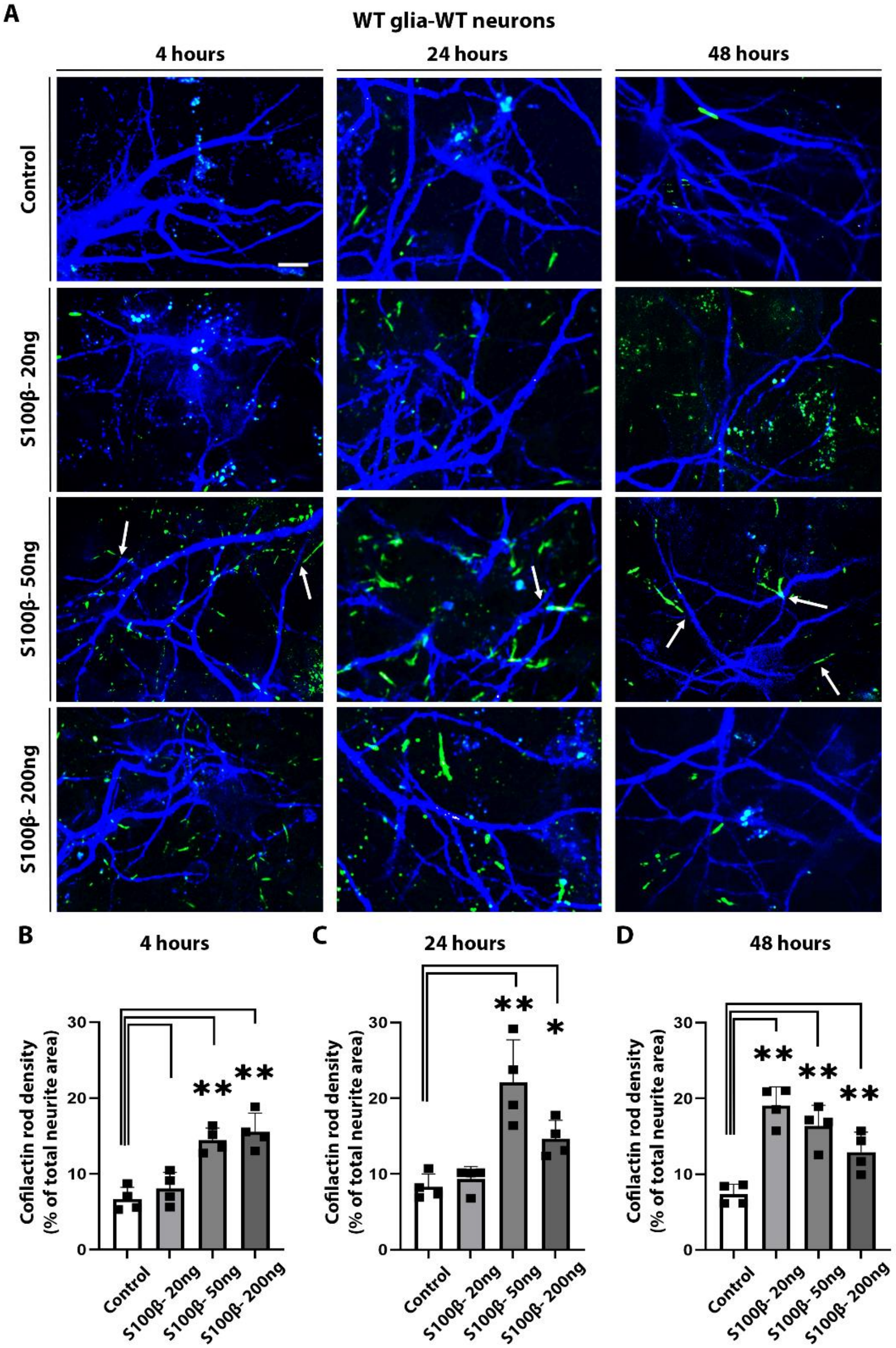

Figure S7, related to Figure 6. Dose- and time- response of S100β on CAR formation in neuron-glia co-cultures.

(A) Photomicrographs of co-cultures immunostained for MAP-2 (blue) and CAR (cofilin-1; green). Arrows identify examples of MAP-2 continuity disrupted by CAR formation. Scale bar = 10 μm. **(B-D)** CAR density expressed as percent of total neurite area. n = 4; *p, 0.05, **p < 0.01 vs. control by one-way ANOVA with Dunnett's test. All data are shown as mean ± SEM.

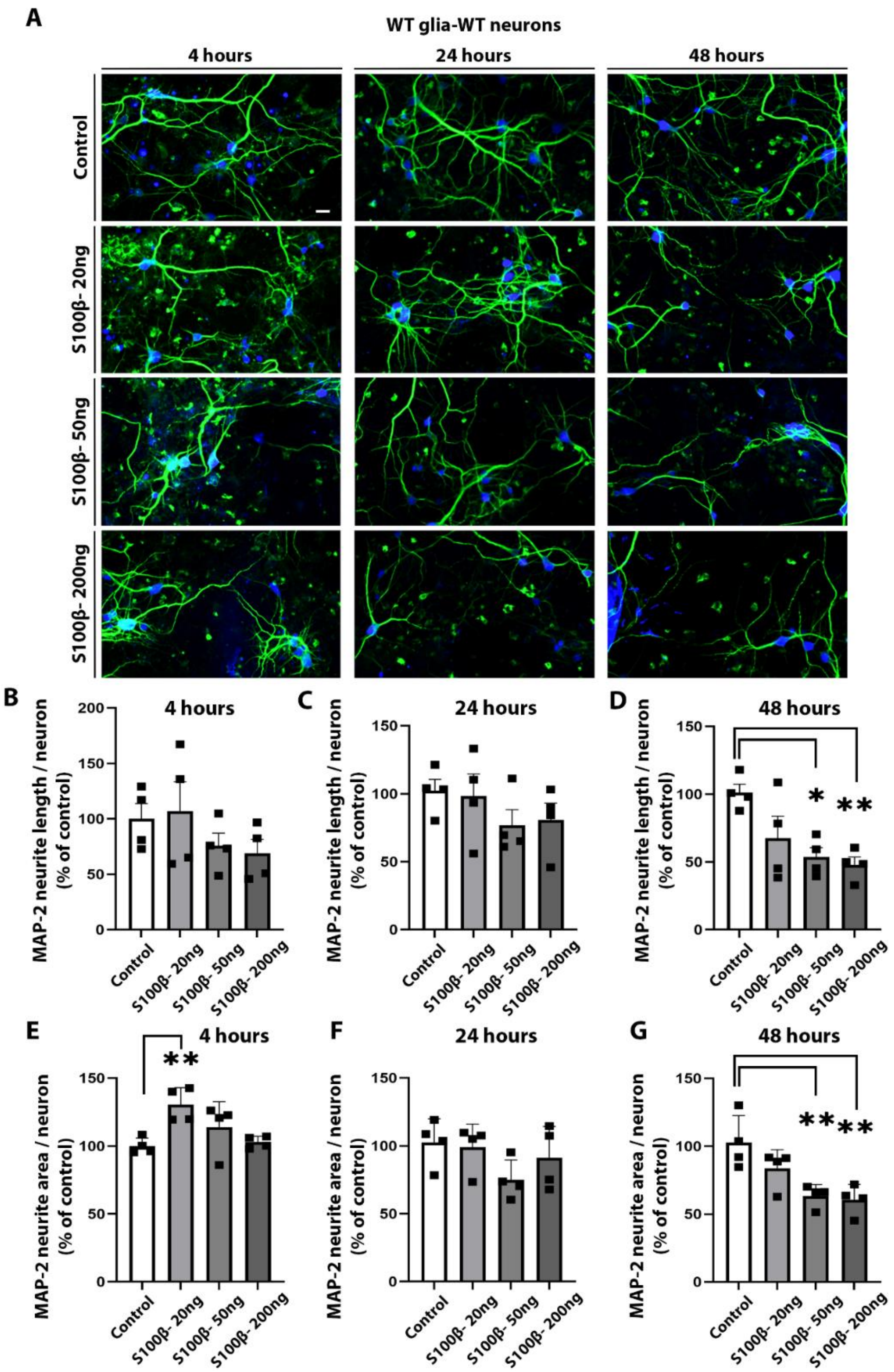

Figure S8, related to Figure 7. Dose- and time-dependent effects of S100β on neurite loss in wild-type neuron-glia co-cultures.

(A) Photomicrographs of co-cultures immunostained for MAP-2 (green) and NeuN (blue). Scale bar = 10 µm. **(B-D)** Neurite loss assessed by MAP-2 length / neuronal nucleus. **(E-F)** Neurite loss assessed by MAP-2 area / neuronal nucleus. Values are expressed relative to control wells prepared in parallel. n = 4; *p, 0.05, **p < 0.01 vs. by one-way ANOVA with Dunnett's test. All data are shown as mean ± SEM.

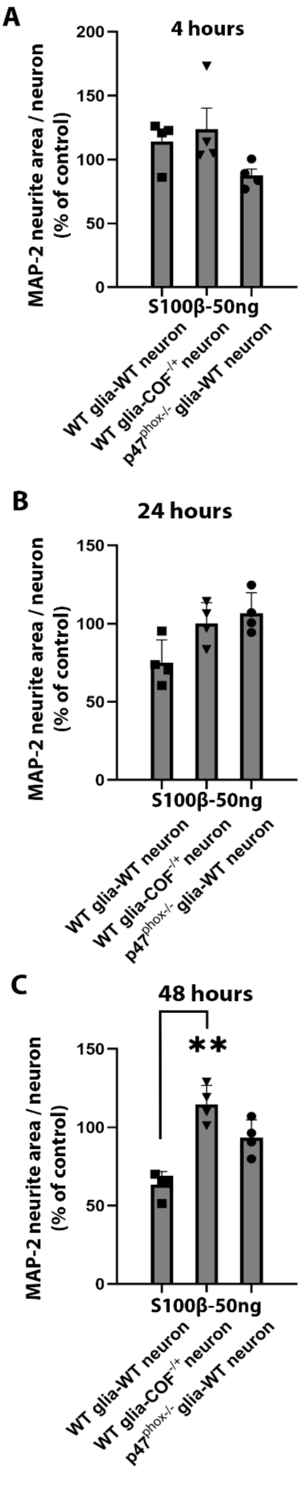

Figure S9, related to Figure 7. Neurite loss in neuron-glia co-cultures of mixed genotypes assessed by MAP-2 area. (A-C) Neurite area measurements corresponding to neurite length assessments in Figure 7. Data are shown as MAP-2 area / neuronal nucleus, expressed relative to the control condition of the respective co-culture type. n = 4; *p, 0.05, **p < 0.01 by one-way ANOVA with Dunnett's test. All data are shown as mean ± SEM.

References

S1. SMi, J., Shaw, A.E., Pak, C.W., Walsh, K.P., Minamide, L.S., Bernstein, B.W., Kuhn, T.B., and Bamburg, J.R. (2013). A genetically encoded reporter for real-time imaging of cofilin-actin rods in living neurons. PloS one *8*, e83609. 10.1371/journal.pone.0083609.
